# Study protocol for a randomized controlled trial of neurofeedback mindfulness in chronic migraines

**DOI:** 10.1016/j.conctc.2024.101362

**Published:** 2024-08-28

**Authors:** Faly Golshan, Rachel Lysenko, Monika Nabi Zade, Parham Alibolandi, Haley Block, Paul Masiowski, Megan E. O'Connell, Gloria Sun, Marla Mickleborough

**Affiliations:** a Department of Psychology & Health Studies, University of Saskatchewan, Canada; b College of Medicine, University of Saskatchewan, Canada

**Keywords:** Neurofeedback, Mindfulness, Chronic migraine, Nonprophylactic treatments, Randomized controlled trials

## Abstract

Chronic migraine is a debilitating headache disorder that is associated with excessive analgesic use. As the long-term use of analgesics could cause additional headaches due to medication overuse, there is a need to probe efficient nonprophylactic alternatives and migraineurs’ long-term adherence to such possible treatments. This protocol investigates the integration of neurofeedback and mindfulness which are the two common nonpharmacological therapies for migraines. We offer the use of portable EEG headbands for easy home-based data collection and consistent data access from researchers. In order to evaluate the efficacy of this recommended intervention, this is a protocol for a randomized control trial with a waitlisted group and an intervention group consisting of a daily attention task. The protocol presents important criteria which should be checked for consistency in longitudinal data collection from adults with chronic migraine.

## 1 Introduction

Migraine is a complex neurological disorder that is comorbid with psychiatric disorders [1,2] or cognitive dysfunction [3,4]. Migraine is the first category of primary headache disorders in the third edition of the International Classification of Headache Disorders [5] and is described based on specific headache characteristics that define subtypes causing mild to severe levels of disability that usually require prophylactic and/or non-prophylactic treatments [6]. Any form of migraine with headaches less than 15 times per month is considered as “episodic migraine”, but if headaches occur on 15 or more days per month for a duration of more than 3 months with at least 8 days/month having features of migraine, then the headaches are categorized as “chronic migraine” which are a bigger cause of disability [5].

Chronic migraine is a form of primary headache disorder, but most of the symptoms overlap with “medication-overuse headache” (MOH), a term generally used for the caused increase in frequency of primary headaches attributed to frequent use of acute or symptomatic medication [7]. Given that long-term pharmacological treatments for migraine could expose the individuals to side effects such as more increased headaches as a function of MOH, it is important to explore the efficacy of non-pharmacological treatments more seriously.

Currently there are many nonpharmacological approaches which are being studied for migraine management, most of which still need to be further explored in terms of their optimization [8]. For example, transcranial magnetic stimulation (TMS) is a procedure during which a magnetic coil is used to influence the electrical activities of the cortex, so the frequency of migraine attacks is reduced, but the available literature review does not support whether this treatment efficiently reduces pain severity or not [9]. At the same time, systematic reviews on other forms of treatments do not provide enough evidence for a decrease in migraine intensity followed by cognitive-behavioural therapy [10] or changes in migraine frequency and medication use followed by acceptance and commitment therapy [11]. Regarding this, the available gap urges more investigation of approaches that not only reduce the frequency and intensity of migraines, but they can also improve individuals’ self-efficacy to manage their headaches with less medical alternatives.

Two other well-known non-pharmacological alternatives for migraine headaches are neurofeedback and mindfulness meditation [12]. Neurofeedback therapy is a non-invasive technique of recording electrical activities of the cortex through EEG and presenting this as feedback to the user in real time while the participant is doing a specific task. Neurofeedback is a subcategory of biofeedback therapy as it engages participants to influence their cortical activities based on the display representing their brainwave activities [13]. At the same time, mindfulness meditation is an evidence-based intervention with practices that specifically concentrate on health improvement. Mindfulness includes states or traits that assist a relaxed non-reactive observation of the mind and bodily functions [14]. Mindfulness technique modifies how one attends to the internal and external factors; the efficacy of this attentional adjustment is found to be beyond a placebo effect or a sham effect [15]. However, there are still questions about the complexity of mindfulness and its relation to the other attention-based practices [17]. A recent investigation has showed that sham mindfulness with some focused-attention techniques might result in improvements in pain threshold similar to mindfulness; nevertheless, the impact on the quality of observing the pain and evaluating its unpleasantness is still significantly greater as a function of mindfulness. Additionally, Davies et al. [16] recommend regular mindfulness practice as a helpful technique for migraine management but they suggest studies that evaluate its efficacy compared to other novel controlled interventions.

In general, the past decade has brought about new research into mindfulness-based treatments as a promising non-pharmacological alternative for migraine management showing that mindfulness could decrease both migraine frequency and intensity [[17], [18], [19], [20], [21]]. Nonetheless, while neurofeedback is a convenient technique for tangible and concrete monitoring of physiological changes throughout a migraine treatment, the studies of neurofeedback and migraine are still few in numbers and most of them have been applied on pediatric populations or in lab-based contexts [[22], [23], [24], [25]]. Studies on either neurofeedback or mindfulness meditation have varied study designs and methodologies yet randomized controlled trials (RCTs) are considered the most appropriate study design for investigating the efficacy of treatments. Yet, more information is needed on how to implement a consistent long-term study of these techniques in the migraine population.

Since it is hard to fully discover if an individual is doing the mindfulness-based practices correctly at a home-based context, real-time EEG feedback could help the participants to learn how to modify their bodily postures and their practices for an optimized practice. So far, no study has ever investigated the efficacy of portable EEG headbands in migraine management and there is a need to see if such emerging technologies are promising or not.

In terms of exploring a new non-pharmacological alternative, the following notes should be critically considered: 1. Cognitive and psychiatric symptoms are associated with migraine headaches [26]; this suggests that alternative treatments should attend to the multifaceted need of adults with chronic migraine for something that improves their quality of life and mental health, not only to cope with pain during an attack, but also to prevent from further attacks through modifying stressors. 2. Since adults with chronic migraine have a higher chance of MOH as the function of frequent intake of analgesics to avoid pain, we need to explore how the suggested alternatives could improve headache sufferers’ pain tolerance and ability to withhold or decrease the overuse of medicine. 3. Most importantly, we need to evaluate practice adherence and feasibility of the alternative with regards to the amount of commitment that participants would have towards the recommended treatment. Both adherence and feasibility are important factors to consider when investigating a non-pharmacological treatment in order to ensure its long-term effectiveness for chronic conditions.

We present our RCT protocol on the efficacy of neurofeedback mindfulness meditation for adults with chronic migraine. In this protocol, we describe a portable EEG headband (MUSE), we address the possible challenges regarding the project launch, data collection, and daily follow up with the participants. The current RCT protocol presents a valuable investigation of a non-pharmacological treatment for chronic migraine for the following reasons: 1. We have taken a novel approach by integrating two common non-pharmacological approaches for migraine i.e., neurofeedback and mindfulness meditation. 2. We introduce a novel controlled intervention that will be discussed in further details. 3. We offer an innovative longitudinal design with direct and consistent follow ups for the whole duration of the study. 4. This study focuses on self-guided mindfulness for naïve meditators who never receive formal training for their practices.

Both mindfulness and neurofeedback techniques have “attentional adjustment” as a key point in common; something that is found to be impaired in migraine headache [[27], [28], [29]]. No previous study has investigated whether the joint techniques could be different from a similar controlled intervention. While it is speculated that neurofeedback mindfulness provides an internal source for the subjects to attend to (e.g., their breath, and their stream of thought) and actively engages them to modulate their cognitive and behavioural responses, our controlled group will also have a level of attentional engagement with their given stimuli. Comparing these two interventions will reveal if migraine management requires a mindfulness routine engagement or if simple attentional adjustments could be sufficient. At the same time, previous literature is suggestive of an interaction between the expectancy and belief in treatments with the reports of improvements by headache sufferers [30]. Hence, it is an important to check whether there is an association between how people benefit from interventions based on their belief in the recommended treatments. Regarding this, in our RCT protocol participants will also be assessed on their belief in the given interventions and how it might impact dependence on medicine intake.

In this longitudinal RCT, we will use a portable Canadian-based EEG headband called MUSE by InteraXon. This headband facilitates data collection in a more convenient way compared to lab-based investigations. Because they can take it home, this headband enables participants to learn about their cortical activities more frequently than when they are asked to come in and wear EEG caps in a lab-based setting. There is a rising interest in the use of wearable interactive devices for telehealth and innovative healthcare support, facilitating a patient-centered health provision and telemonitoring [31]. As a validated portable EEG device [32], MUSE is designed to enable participants with a report of their attention based on their brainwaves during different relaxation techniques; such devices are found helpful with moment-to-moment reports of the electrical activities in different conditions [31]. This device is known as a low-cost EEG device (less than 500 CAD) [31,33] and enables a more convenient remote access to participants’ practices via a suggested platform, called MUSE Connect, where the practice history of the individuals is displayed.

## 2 Hypotheses

The main goal of the presented RCT protocol is to evaluate how 8 weeks of neurofeedback mindfulness using a wearable EEG headband could moderate headache experience (including frequency and intensity), headache management self-efficacy, psychiatric symptoms (i.e., anxiety and depression) and dependence on analgesics for adults with chronic migraine who are also identified as naïve meditators. The specific measures are described in more detail below. The following hypotheses are tested in this RCT.

1. After completing the intervention, neurofeedback mindfulness will improve headache experience across time via:
  a. Decreased migraine disability
  b. Decreased migraine intensity
  c. Increased headache management self-efficacy
    a. Decreased anxiety
    b. Decreased depression
    c. Decreased level of dependence on medication.
2. Participants in the neurofeedback mindfulness group will have significantly lower scores in migraine disability and intensity, and significantly higher scores in headache management self-efficacy when compared to the attention control group and the waitlisted group.
3. Participants in the neurofeedback mindfulness group will have lower anxiety and lower depression scores when compared to the attention control group and the waitlisted group.
4. Neurofeedback mindfulness participants will have a significant decrease in the level of dependence on medication, compared to those in the controlled intervention and waitlisted groups.
5. There will be no impact of participants' belief in meditation on any of the measured criteria (e.g., migraine characteristics and psychiatric symptoms) across time.

## 3 Methods

### 3.1 RCT design

This protocol proposes a longitudinal RCT to compare neurofeedback mindfulness with two control groups: a simple attention intervention and a waitlisted group. Similar to the previous RCTs in mindfulness and migraine, the duration of this trial is scheduled for 8 weeks [20,[34], [35], [36]] with a 10-min practice per day [17]. This study protocol includes two major phases: Phase I for RCT design preparation and Phase II for data collection.

During Phase I, plans are made for participant recruitment and ensuring that all ethical concerns are met. Since the portable EEG is connected to the third-party app (also named MUSE) on smartphone, plans should be made to ensure that no personal identifiable details are shared on the app. Importantly, the researchers are required to monitor the participants' activities on the MUSE app every day. Due to these factors, we will equip all the participants with generic coded emails that are de-identified for completing the surveys on SurveyMonkey and registration of participants on the MUSE app. The generic emails are all connected to our lab's email so that the researchers can verify and proceed with MUSE app's registration process for each participant. Prior to data collection, it is necessary to sign up for a platform, called MUSE Connect, which provides a list of all the participants' practices for daily monitoring of the groups' exercises. The university-based generic emails are available throughout all the study and their codes will be used to refer to the participants. Phase II of this study includes posting announcements seeking adults with chronic migraine in neurology clinics, contacting volunteers, accepting participants, scheduling device delivery, initial device and app set up, data collection monitoring, and daily reminders for the tasks to the participants.

### 3.2 Study procedure

Participant recruitment occurs through neurology clinics and walk-in clinics in Saskatoon, Canada. The posters will be emailed to individuals who have been referred to the clinics. Once the volunteers email the lab, the initial questionnaire and consent forms will be shared with them. The initial questionnaire includes 45 open-ended and multiple-choice questions (estimated to take 26 min to complete on average) about descriptive information, migraine characteristics and history of the participants as well as their background experience with meditation and whether they believe that meditation could help their migraine headaches. The participants’ history of taking analgesics and acute medications will be assessed based on severity of dependence scale (SDS) [37].

### 3.3 Eligibility screening

All the participants will be screened for eligibility before an invitation is sent to them. To be eligible, the participants should 1. be over 18 years of age, 2. have a diagnosis of chronic migraine from a clinician or have met the criteria for a chronic migraine diagnosis based on ICHD-3 [5], 3. have no frequent background experience of meditation, 4. reside in Saskatoon or be able to receive the device from Saskatoon and the communities nearby, 5. have a smartphone and internet connection for accessing the MUSE app. The exclusion criteria for this study consist of.

1. Comorbidity of Raynaud's syndrome or diabetes,
2. Plans to start a new preventative migraine treatment.

After checking eligibility criteria, the participants will be randomly allocated in the three arms of the study: the neurofeedback mindfulness, the controlled intervention, and the waitlisted group. The participants will receive an invitation letter with a description of their task, the PDF form of their consent, and the contact information of the researcher in charge of the device delivery.

### 3.4 Initial meeting

For the two intervention groups, an online meeting will be scheduled (estimated 30–60 min) after the participants receive the device. During the session, the following steps will be checked.

• A brief introduction to the study goals and tasks
• Request for a verbal consent to proceed with the meeting
• Help with setting up and wearing the headband
• Downloading and signing into the MUSE app
• Tutorials on the assigned practice, and device calibration

The participants are required to complete the pre-intervention questionnaire on the day of the initial meeting.

### 3.5 Intervention follow up

**Fig. 1:**
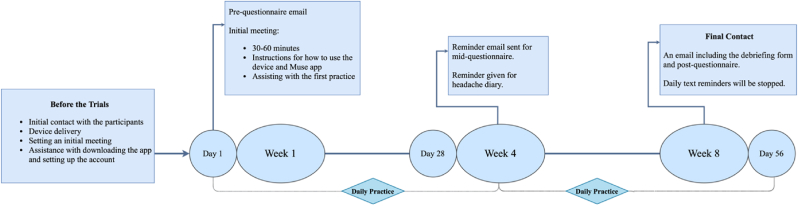
Flowchart for intervention procedure in the study.

Except for the waitlisted group, participants will receive daily reminders from a researcher scheduled at 8:00 p.m. They will be required to respond to the researcher by sending “1” for a complete session. All the participants' practices will be monitored daily via MUSE Connect. This platform displays information about the percentage of relaxed or active states and duration of the practice. In case of a pause for more than three days, the researcher will send a follow up with the participant. On Week 4 and Week 8 of the study, the researcher will send participants mid- and post-intervention questionnaires. In the event of having issues with the device, a replacement EEG headband will be scheduled for delivery upon participants’ availability (Fig. 1).

The waitlisted group are also required to complete the questionnaires on Week 1, Week 4, Week 8 after being invited to the study and the MUSE device will be delivered to them after completing all three of their questionnaires. In the meantime, they will fill out headache diaries regarding their headache experience. The diaries are accessible via SurveyMonkey and include 24 multiple choice questions (9 min on average) about subjects’ recent headache experience.

### 3.6 The assigned tasks

In this study, the participants will be randomly allocated in three groups of neurofeedback-mindfulness, the controlled intervention, and waitlisted groups. The allocation will be done using stratified randomization to ensure equal distribution (1:1:1) among groups.

1. The neurofeedback mindfulness group
  This group is asked to sit comfortably in a quiet area and do one of ten beginner self-guided mindfulness sessions on mind-body scanning techniques from within the MUSE app. Each session includes a brief explanation for 2–3 min before the practice. The participants repeat the same sessions throughout the intervention.
  1. Introduction to mindfulness: a brief review of mindfulness as a nonjudgmental observation of body and mind.
  2. Training a puppy: how to monitor emotions and control reactions to different states of mind.
  3. Sensation of breath: the importance of mindful breathing techniques.
  4. Counting breaths: how to inhale and exhale properly.
  5. Sitting comfortably: how to modify positions for mindfulness practices.
  6. Finding your soundscapes: monitoring the states of mind and how they are associated with the given neurofeedback sounds.
  7. Dealing with distraction: describing nonreactivity and acceptance of different states of mind.
  8. Working with discomfort: how to mindfully adjust the states of mind in unfavourable conditions.
  9. Lowering the bar: Moderating expectations from practices and accept the present state of mind without changing it.
  10. Bridging to daily life: bringing attention to how the state of acceptance could be conceptualized in everyday routines.

Once the participants start their practices, they receive sounds for their real-time brain activities: thunderstorm for an active state, ocean waves for a neutral state, and birds chirping for a relaxed state. For each 5 s of consistent relaxed state, the participants are reinforced with points.

2. The attention control group

The participants in the attention control group are assigned to have an attention routine in a relaxed position. They are required to sit comfortably and relax in a quiet area to put on their MUSE headband, set it up, and use the same practices on MUSE app but they will mute all the neurofeedback sounds and instructions. Instead, on their smartphone the participants will check their emails, the news, or their preferred social media platform for 10 min. Once the session is over, the participants receive the graphical feedback of their brain activities. The feedback includes a timeline indicating their brain states (active, or relaxed) during the 10-min session.

3. The waitlisted group

**Fig. 2:**
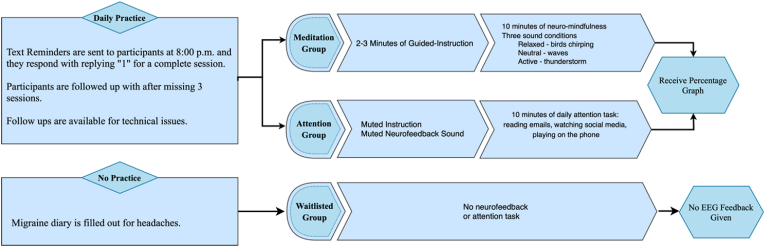
The assigned tasks for each of the study groups.

The participants in this group are informed about being appointed in the waitlisted group and that we will only be collecting headache data from them for 8 weeks. They will complete the questionnaires for three rounds (i.e., Week 1, Week 4, and Week 8) and information about their headaches will be collected via the headache diaries. After 8 weeks, they will receive the MUSE device and will be offered a complementary session on how to use the device for their personal use. A debriefing form will be emailed to all the participants after completion of the study (Fig. 2).

### 3.7 Incentives

Since this study is demanding and expects a high amount of commitment, we appreciate participants’ collaboration by allowing them to keep their MUSE headband at the end of the study after completion of the data collection. The participants who withdraw before completion are also allowed to keep their device.

### 3.8 Blinding

Due to the nature of this RCT design, it is not possible for participants to be blinded to their group assignment. However, the subjects in all three groups will be coded and de-identified after initial meeting for the whole duration of data collection and data analysis procedure. They are requested to keep their contact by mentioning their given codes and fill out the questionnaires by submitting the USASK coded emails instead of their personal emails.

### 3.9 Participants

**Fig. 3:**
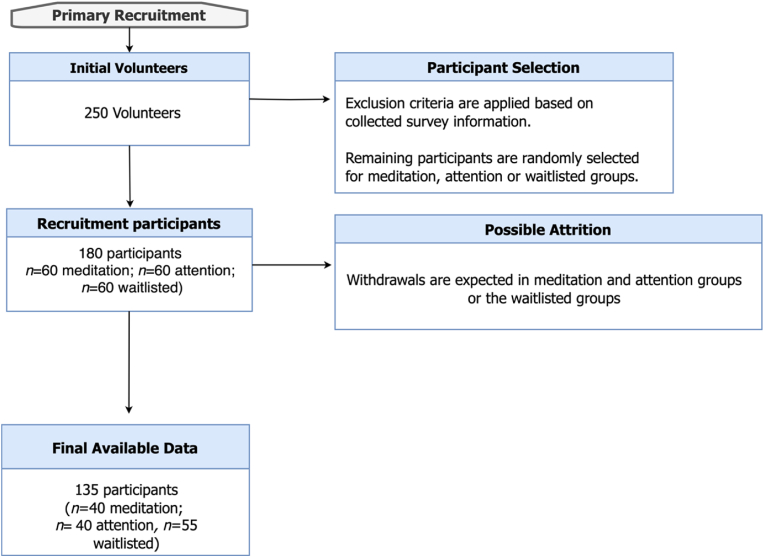
The flowchart for Participant Recruitment.

According to A priori G*power software calculation for this study considering both between-subject and within-subject effects, a sample size of 66 is expected to detect significance level of 0.05 with 95 % power through our planned statistical analysis test, i.e., Repeated Measures ANOVA. We aim to look at the effect of time by comparing the baseline with mid-/post intervention reports of the participants for each of the six measurements independently; moreover, we will compare the interaction of time*groups separately for each of our six scales. We will initially allocate 180 participants; yet with reference to the previous studies [22], we could expect up to 50 % of attrition. After applying inclusion and exclusion criteria, participants will be selected for either the meditation, the control, or the waitlisted group. We speculate a higher number of attritions in the meditation and control groups regarding the complexity of the given tasks as compared to the waitlisted group. Fig. 3 provides a flowchart for the number of expected participants at each stage of the study.

### 3.10 Attrition

Due to the high demanding nature of chronic migraine and concerns with balancing expectations and time commitment for adults with chronic migraines, a high rate of attrition is expected. Previous non-migraine related RCTs have had attrition rates as high as 48 % [38]. With regards to previous similar studies on long-term use of neurofeedback in migraine populations with 75 % of attrition [22], we will speculate a high rate of attrition (50 %) for 8 weeks of joint neurofeedback mindfulness interventions.

### 3.11 The instruments

In this study, six measurements are given to participants at three time points during the study: before the intervention (Week 1), during the intervention (Week 4) and after completing the intervention (Week 8). On average, the total amount of time to complete six questionnaires is estimated around 13 min. These measurements (described in detail below) collect information about migraine disability (MIDAS), headache intensity (HIT-6), headache management self-efficacy (HMSE), anxiety (BAI), depression (CES-D), and severity of dependence on medications (SDS).

1 Migraine Disability Assessment Scale (MIDAS)

This 5-item instrument focuses on the number of headache-impacted missing days in the migraine population. MIDAS is shown to have significantly high internal consistency scores in United States (Cronbach's α = 0.76) and the UK (Cronbach's α = 0.73) [39].

2 Headache Impact Test-short form (HIT-6)

This 6-item scale collects information about the intensity of the headache attacks with high reliability of 0.90 Cronbach's ***α*** ([40]). This measurement is a short and sound psychometrical tool to assess how participants evaluate their headache severity.

3. Headache Management Self-Efficacy Scale (HMSE)

This measurement is a 25-item scale that measures individuals' perceived level of coping and headache-related disability management with an excellent internal consistency (Cronbach's α = 0.90) [41]. The HMSE score predicts headache-related disability, as shown in MIDAS, regardless of the headache intensity, as usually shown in HIT-6 [41].

4 BECK Anxiety Inventory (BAI)

This 21-item instrument is a self-report of physical and cognitive symptoms of anxiety on and is validated with a moderate correlation with the revised Hamilton Anxiety Rating Scale (*r* = 0.51)([42]). Internal consistency of this measure is significantly high (Cronbach's α = 0.92) and test-retest reliability in a 1-week period was also acceptable (Cronbach's α = 0.75) [42].

5. The Center for Epidemiologic Studies Depression (CES-D)

This validated 20-item scale is a short self-report of the original version with 300 items [43]. The internal consistency of this instrument is high (Cronbach's α = 0.85–0.90) and test re-test reliability is also acceptable (0.45–0.70) [39].

6. Severity of Dependence Scale (SDS)

This scale includes 5 questions that originally evaluate participants' level of dependence on different substances [37,44]. It has recently been validated to be effective with assessing dependence-like behaviours in subjects with MOH [45] with a high internal reliability (α = 0.72). This scale will be a reliable tool to assess participants’ changes in tolerance and need for increased amount of the medication to manage their headaches.

### 3.12 Statistical methods

This protocol is primarily based on comparing the baseline information of the participants with their information on Weeks 4 & 8 of their intervention. With regards to this, we will use Repeated Measures ANOVA for comparing the effect of time on the participants' changes in chronic headache characteristics, psychiatric symptoms, and medicine intake. Additionally, we will compare the interaction effect of time* groups to discover which of the three groups would show most significant changes across time as measured independently for each of the study's scales.

For each of the task-based groups, we will explore the total missed days and attrition at each of the task groups to discover the level of adherence for the two task-based groups. The diaries will also be compared across time to discover how participants manage their headaches via different alternatives and whether there is a change in their trend of headache medicine intake.

Finally, the EEG data analysis will be applied for within-subject and between subject comparisons to determine whether there is a change in adults with chronic migraine's peak alpha frequency over time and during their headache experiences as a function of their practices.

## 4 Discussion

This RCT protocol has a pioneering concept of introducing a new self-guided neurofeedback mindfulness meditation in adults with chronic migraine compared to a novel controlled intervention and a waitlisted group. Our study protocol includes details on the most important challenges, specifically data collection and issues with confidentiality while using a third-party app. Most importantly, we need to evaluate how to keep participants in a consistent and thorough routine. To motivate participants, we have recommended manual text reminders which support participants with easy access to troubleshooting. Since chronic migraine a lifelong, it is important to take measures for both respecting their choice of withdrawal, as well as reinforcing participants' adherence throughout the study. The recommended protocol is designed to keep a consistent practice balance for adults with chronic migraine to collect information about the effectiveness of neurofeedback mindfulness meditation. We also introduce portable EEG headband as a means of collecting daily information from the participants’ practices.

## Ethics approval

This study is approved by the Behavioural Research Ethics Board at the University of Saskatchewan (Beh-REB 1986). The researchers are all trained by the Tri-Council Policy Statement: Ethical Conduct for Research Involving Humans (TCPS 2).

## Funding

This 10.13039/100014144RCT research is funded by 10.13039/501100000106Saskatchewan Health Research Foundation (10.13039/501100000106SHRF) granted to Dr. Marla Mickleborough in 2019 (GRANT ID Number 423629) at the 10.13039/100008920University of Saskatchewan.

## CRediT authorship contribution statement

**Faly Golshan:** Writing – review & editing, Writing – original draft, Methodology, Conceptualization. **Rachel Lysenko:** Writing – review & editing, Visualization. **Monika Nabi Zade:** Writing – review & editing. **Parham Alibolandi:** Writing – review & editing. **Haley Block:** Supervision. **Paul Masiowski:** Supervision. **Megan E. O'Connell:** Supervision. **Gloria Sun:** Conceptualization. **Marla Mickleborough:** Writing – review & editing, Funding acquisition, Conceptualization.

## Declaration of competing interest

The authors declare that they have no known competing financial interests or personal relationships that could have appeared to influence the work reported in this paper.

## Data Availability

No data was used for the research described in the article.

## References

[1] Dodick D.W. (2018 May 1). A phase-by-phase review of migraine pathophysiology. Headache.

[2] Wells R.E., Seng E.K., Edwards R.R., Victorson D.E., Pierce C.R., Rosenberg L. (2020). Expert Review of Neurotherapeutics.

[3] Fong C.Y., Law W.H.C., Braithwaite J., Mazaheri A. (2020 Jan 1). Differences in early and late pattern-onset visual-evoked potentials between self- reported migraineurs and controls. Neuroimage Clin.

[4] Chen G., Li Y., Dong Z., Wang R., Zhao D., Obeso I. (2020). Response inhibition alterations in migraine: evidence from event-related potentials and evoked oscillations. J. Headache Pain.

[5] Olesen J. (2018). Cephalalgia.

[6] Dodick D.W. (2018 May 1). A phase-by-phase review of migraine pathophysiology. Headache.

[7] Weatherall M.W. (2015). The diagnosis and treatment of chronic migraine. Ther Adv Chronic Dis.

[8] Haghdoost F., Togha M. (2022 Nov 23). Migraine management: non-pharmacological points for patients and health care professionals. Open Med..

[9] Zhong J., Lan W., Feng Y., Yu L., Xiao R., Shen Y., Zou Z., Hou X. (2022 Nov 24). Efficacy of repetitive transcranial magnetic stimulation on chronic migraine: a meta-analysis. Front. Neurol..

[10] Bae J.Y., Sung H.K., Kwon N.Y., Go H.Y., Kim T.J., Shin S.M., Lee S. (2021 Dec 28). Cognitive behavioral therapy for migraine headache: a systematic review and meta-analysis. Medicina.

[11] Polk A.N., Smitherman T.A. (2023 Oct). A meta-analytic review of acceptance-based interventions for migraine. Headache.

[12] Dodick D.W. (2018).

[13] Hershaw J.N., Hill-Pearson C.A., Arango J.I., Souvignier A.R., Pazdan R.M. (2020 Mar 2). Semi-automated neurofeedback therapy for persistent postconcussive symptoms in a military clinical setting: a feasibility study. Mil. Med..

[14] Kabat-Zinn J. (1982). An outpatient program in behavioral medicine for chronic pain patients based on the practice of mindfulness meditation:. Theoretical Considerations and Preliminary Results.

[15] Zeidan F., Emerson N.M., Farris S.R., Ray J.N., Jung Y., McHaffie J.G. (2015). Mindfulness meditation-based pain relief employs different neural mechanisms than placebo and sham mindfulness meditation-induced analgesia. J. Neurosci..

[16] Davies J.N., Sharpe L., Day M.A., Colagiuri B. (2021 Jul 1). Mindfulness-based analgesia or placebo effect? The development and evaluation of a sham mindfulness intervention for acute experimental pain. Psychosom. Med..

[17] Sansone E., Grazzi L., Raggi A., Leonardi M., D'Amico D. (2020 Dec 1). Mindfulness as an add-on treatment for patients with chronic migraine and medication overuse: a preliminary analysis. Neurol. Sci..

[18] Tonelli M.E., Wachholtz A.B. (2014). Meditation-based treatment yielding immediate relief for meditation-naïve migraineurs. Pain Management Nursing [Internet].

[19] Wachholtz A., Vohra R., Metzger A. (2019). A reanalysis of a randomized trial on meditation for migraine headaches: distraction is not enough but meditation takes time. Compl. Ther. Med..

[20] Wells R.E., Burch R., Paulsen R.H., Wayne P.M., Houle T.T., Loder E. (2014). Meditation for migraines: a pilot randomized controlled trial. Headache.

[21] Wells R.E., Loder E. (2012). Mind/Body and behavioral treatments: the evidence and approach. Headache.

[22] Connelly M., Boorigie M., McCabe K. (2023 Feb 1). Acceptability and tolerability of extended reality relaxation training with and without wearable neurofeedback in pediatric migraine. Children.

[23] Stokes D.A., Lappin M.S. (2010). Neurofeedback and biofeedback with 37 migraineurs: a clinical outcome study. Behav. Brain Funct..

[24] Walker J.E. (2011). QEEG-guided neurofeedback for recurrent migraine headaches. Clin. EEG Neurosci..

[25] Siniatchkin M., Kropp P., Gerber W.D. (2003 Sep). What kind of habituation is impaired in migraine patients?. Cephalalgia.

[26] Lipton R.B., Seng E.K., Chu M.K., Reed M.L., Fanning K.M., Adams A.M. (2020 Sep 1). The effect of psychiatric comorbidities on headache-related disability in migraine: results from the chronic migraine epidemiology and outcomes (CaMEO) study. Headache.

[27] Golshan F., Moss D., Sun G., Krigolson O., Cruz M.T., Loehr J. (2022 Sep 1). ERP evidence of heightened attentional response to visual stimuli in migraine headache disorders. Exp. Brain Res..

[28] Mickleborough M.J.S., Truong G., Handy T.C. (2011). Top-down control of visual cortex in migraine populations. Neuropsychologia [Internet].

[29] Mickleborough M.J.S., Ekstrand C., Gould L., Lorentz E.J., Ellchuk T., Babyn P. (2016). Attentional network differences between migraineurs and non-migraine controls: fMRI evidence. Brain Topogr..

[30] Autret A., Valade D., Debiais S. (2012). Placebo and other psychological interactions in headache treatment. J. Headache Pain.

[31] Hunkin H., King D.L., Zajac I.T. (2019).

[32] Krigolson O.E., Williams C.C., Norton A., Hassall C.D., Colino F.L. (2017 Mar 10). Choosing MUSE: validation of a low-cost, portable EEG system for ERP research. Front. Neurosci..

[33] Bird J.J., Manso L.J., Ribeiro E.P., Ekart A., Faria D.R. (2018). A study on mental state classification using EEG-based brain-machine interface;. A Study on Mental State Classification using EEG-based Brain-Machine Interface.

[34] Seng E.K., Singer A.B., Metts C., Grinberg A.S., Patel Z.S., Marzouk M. (2019). Does mindfulness-based cognitive therapy for migraine reduce migraine-related disability in people with episodic and chronic migraine? A phase 2b pilot randomized clinical trial. Headache.

[35] Seminowicz D.A., Burrowes S.A.B., Kearson A., Zhang J., Krimmel S.R., Samawi L. (2020 Aug 1). Enhanced mindfulness-based stress reduction in episodic migraine: a randomized clinical trial with magnetic resonance imaging outcomes. Pain.

[36] Napadow V. (2020).

[37] Lundqvist C., Benth J.Š., Grande R.B., Aaseth K., Russell M.B. (2011 Mar). An adapted Severity of Dependence Scale is valid for the detection of medication overuse: the Akershus study of chronic headache. Eur. J. Neurol..

[38] Dumville J.C., Torgerson D.J., Hewitt C.E. (2006). Analysis and comment [internet]. BMJ.

[39] Stewart W.F., Lipton R.B., Dowson A.J., Sawyer J.M. (2001 Mar). Development and testing of the migraine disability assessment (MIDAS) questionnaire to assess headache-related disability. Neurology.

[40] Kosinski M, Bayliss M, Bjorner J, Ware Jr J, Garber W, Batenhorst A, et al. A six-item short-form survey for measuring headache impact: The HIT-6ä. 10.1023/a:1026119331193 14651415

[41] French D.J., Holroyd K.A., Pinell C., Malinoski P.T., O'Donnell F., Hill K.R. (2000). Perceived self-efficacy and headache-related disability. Headache.

[42] Beck A. (1988).

[43] Radloff L.S. (1977). The CES-D scale:. A Self-Report Depression Scale for Research in the General Population.

[44] Gossop M., Darke S., Griffiths P., Hando J., Powis B., Hall W. (1995). The Severity of Dependence Scale (SDS): psychometric properties of the SDS in English and Australian samples of heroin, cocaine and amphetamine users. Addiction.

[45] Lundqvist C., Gossop M., Russell M.B., Straand J., Kristoffersen E.S. (2019 Sep 1). Severity of analgesic dependence and medication-overuse headache. J. Addiction Med..

